# Preliminary Case–Control Study of Antibody Response to Vaccines in Children on bDMARDs

**DOI:** 10.3390/children12111526

**Published:** 2025-11-11

**Authors:** Asuman Demirbuğa, Elif Dede, Deniz Bahar Akgün Karapınar, Özge Kaba, Neslihan Mete Atasever, Mehmet Akif Durmuş, Mustafa Önel, Ali Ağaçfidan, Ayper Somer, Selda Hançerli Törün

**Affiliations:** 1 Pediatric Infectious Diseases, Mardin Training and Research Hospital, 47100 Mardin, Türkiye; 2 Pediatric Infectious Diseases, Istanbul Faculty of Medicine, Istanbul University, 34093 Istanbul, Türkiye; elif.dede.89@istanbul.edu.tr (E.D.); neslihan.meteatasever@ist.edu.tr (N.M.A.); somer@istanbul.edu.tr (A.S.); selda.hancerli@istanbul.edu.tr (S.H.T.); 3 Department of Medical Microbiology, Istanbul Faculty of Medicine, Istanbul University, 34093 Istanbul, Türkiye; akgund@istanbul.edu.tr (D.B.A.K.); onelm@istanbul.edu.tr (M.Ö.); ali.agacfidan@istanbul.edu.tr (A.A.); 4 Pediatric Infectious Diseases, Başakşehir Çam ve Sakura City Hospital, 34480 Istanbul, Türkiye; ozge.kaba@istanbul.edu.tr; 5 Department of Medical Microbiology, Başakşehir Çam ve Sakura City Hospital, 34480 Istanbul, Türkiye; drmehmetakifdurmus@gmail.com

**Keywords:** anti-HbsAg, anti-PCP, anti-measles IgG, bDMARD, pediatric rheumatic disease

## Abstract

**Highlights:**

**What are the main findings?**

- A subset of patients exhibited low baseline hepatitis B and measles antibody titers even before the initiation of bDMARD therapy.
- During follow-up, a measurable decline in measles antibody levels was observed in a small number of patients, while protective titers for pneumococcus and hepatitis B were largely preserved.

**What are the implications of the main findings?**

- The study highlights the importance of screening vaccine antibody levels before and during bDMARD therapy to detect children at risk of waning immunity.
- Implementing tailored vaccination and timely booster strategies—particularly for measles and hepatitis B—may enhance long-term protection in immunosuppressed pediatric patients.
- This work contributes novel pediatric data on vaccine responsiveness under biologic therapy, emphasizing the need for routine immunological monitoring in clinical practice.

**Abstract:**

**Background/Objectives:** Patients with rheumatic diseases have an increased burden of infection owing to biological disease-modifying antirheumatic drug (bDMARD) therapy. Therefore, vaccination is crucial for the prevention of infection in these patients. In this case–control study, we aimed to evaluate vaccine response to hepatitis B, pneumococcus, and measles using antibody titers in patients undergoing biological therapy. **Methods:** This study included 16 patients aged 5–18 years of age who received bDMARD treatment and 20 healthy controls. Serum samples of the patients were collected at baseline and subsequently on the 3rd and 6th months after bDMARD therapy, and IgG antibodies against pneumococcal capsular polysaccharide antigen (PCP), measles, and hepatitis B were measured. **Results:** There were no statistically significant differences in mean anti-HBsAg, anti-PCP, and anti-measles antibody titers between the study and control groups. The percentages of patients with anti-HbsAg, anti-PCP, and anti-measles protective antibodies were 68.8% (n = 11/16), 100% (n = 16/16), and 56.25% (n = 9/16), respectively. There were no statistically significant differences in the mean antibody titers at baseline and 3rd month. Only the anti-measles IgG titer level decreased below 200 (mIU/mL) in one patient in the 3rd month and in two patients in the 6th month. **Conclusions:** Patients with low or declining hepatitis B and measles antibody titers before or during bDMARD treatment may require close monitoring to ensure adequate protection against vaccine-preventable diseases. Regular screening and follow-up are essential in this patient population.

## 1. Introduction

Patients with rheumatic diseases have an increased burden of infections, which can be attributed to the underlying immune dysregulation or the immunosuppressive effects of biological disease-modifying antirheumatic drug therapy (bDMARD) [1]. bDMARDs are immunomodulatory agents and classes of drugs are indicated for the treatment of inflammatory rheumatic diseases and are typically used after the failure of conventional therapies. Agents such as tumor necrosis factor (TNF) inhibitors (e.g., infliximab, adalimumab, etanercept), interleukin-6 inhibitors (e.g., tocilizumab), T cell co-stimulation modulators (e.g., abatacept), and B cell–depleting agents (e.g., rituximab) exert selective effects on cytokine signaling pathways. While these therapies improve disease control, they may also compromise immune defense mechanisms [2]. The current “treat-to-target” strategy promotes intensive immunosuppressive therapy to achieve remission. However, this approach also emphasizes the importance of preventive strategies—including vaccination—to mitigate infection risks in immunosuppressed children [3].

At the same time, this approach highlights the importance of preventive strategies—including vaccination—to mitigate infection risks in immunosuppressed children. The European League Against Rheumatism (EULAR) and the Pediatric Rheumatology European Society (PRES) both emphasize that children with autoimmune and inflammatory diseases should complete recommended immunizations before starting immunosuppressive therapy whenever possible and should undergo regular assessment of vaccine-induced immunity during treatment [3,4].

Among vaccine-preventable infections, hepatitis B and measles are of particular concern in immunosuppressed children. Hepatitis B virus reactivation has been reported in patients receiving immunosuppressive or biologic therapy, leading to potentially severe outcomes such as fulminant hepatitis. Similarly, measles can cause prolonged viral shedding, severe pneumonia, or encephalitis in immunocompromised hosts, and outbreaks have been increasingly reported in regions with declining vaccine coverage. Monitoring immunity to these infections is therefore critical in pediatric patients treated with bDMARDs.

A previous study demonstrated that vaccine-induced antibody responses were lower in children with juvenile idiopathic arthritis than in healthy controls, likely reflecting incomplete vaccination or the use of immunosuppressive drugs such as biologic agents [4]. There are limited data on immune responses to hepatitis B, pneumococcal, and measles vaccines in children with rheumatic diseases receiving bDMARD therapy [5,6,7,8].

This case–control study aimed to evaluate antibody titers against hepatitis B, pneumococcus, and measles in children receiving bDMARD therapy compared with healthy controls. We hypothesized that, despite complete vaccination, some patients would display suboptimal baseline immunity and a potential decline in antibody titers during follow-up.

## 2. Materials and Methods

This prospective case–control study was conducted between September 2021 and April 2022, at Istanbul University, Istanbul Faculty of Medicine, Türkiye. Pediatric patients aged 5–18 years diagnosed with rheumatic diseases who were scheduled to start bDMARD therapy were enrolled. All patients had previously received conventional treatments (prednisolone, methotrexate, colchicine, or non-steroidal anti-inflammatory drugs) without sufficient clinical response. The control group comprised 20 age- and sex-matched healthy children who attended the hospital for routine health check-ups and had no chronic illnesses or ongoing immunosuppressive therapy. All participants received two doses of MMR (Measles, Mumps, Rubella), three doses of hepatitis B, and four doses of pneumococcal 13-valent conjugate vaccine (PCV13) vaccines. Children whose primary vaccinations were completed according to Public Health Organization recommendations were included in this study. The vaccination status was assessed by checking the vaccination card (manual or e-card). Children with incomplete vaccination records, chronic infections (e.g., hepatitis B, hepatitis C, or HIV), or missing clinical data were excluded. Demographic and clinical features of the patients were prospectively evaluated using their medical records.

### 2.1. Sample Collection and Detection of PCP–Measles–Hepatitis B Specific IgG Antibodies

Serum samples were collected from patients before and at 3 and 6 months after the initiation of bDMARD therapy and stored at −20 °C until analysis. Specific IgG antibodies against pneumococcal capsular polysaccharide antigen (PCP), measles, and hepatitis B were measured using enzyme-linked immunosorbent assay (ELISA). All samples were analyzed in duplicate, and inter-assay and intra-assay variation was monitored using manufacturer-provided control sera. Details of the commercial kits and analyzers used for antibody detection are provided in the Supplementary Material (Table S1).

For measles, IgG values < 150 and >200 mIU/mL were considered seronegative and seropositive, respectively; values between 150 and 200 mIU/mL were considered equivocal. The seroprotection cutoff was defined as >120 mIU/mL, based on the World Health Organization (WHO) 3rd International Standard (NIBSC 97/648) [9,10]. The anti-PCP IgG kit measures antibody responses to 23 pneumococcal polysaccharides representing approximately 80% of virulent Streptococcus pneumoniae serotypes. The assay quantification range was 3.3–270 mg/dL. The protective anti-HBs level was defined as ≥10 mIU/mL.

### 2.2. Statistical Analysis

Statistical data were analyzed using SPSS version 21 (SPSS Inc., Chicago, IL, USA). Values for numerical variables are presented as the mean ± standard deviation, depending on the normal distribution. Categorical variables were presented as numbers and percentages. The conformity of the data to the normal distribution was evaluated using the Shapiro–Wilk test. All antibody titer data were tested for normal distribution before statistical analysis. Comparisons between groups (patients vs. controls) were performed using the independent-samples *t*-test for continuous variables and the χ^2^ test or Fisher’s exact test for categorical variables. Temporal changes in antibody titers across three time points (baseline, 3rd month, and 6th month) were evaluated descriptively using mean ± SD and 95% confidence intervals (CI) to illustrate variability. Given the small sample size, no correction for multiple comparisons was applied, and the results should be interpreted as exploratory. Statistical significance was set at *p* < 0.05. Missing follow-up data from patients who did not return for sampling were excluded from longitudinal analyses.

### 2.3. Ethics Committee Approval

This study was approved by the Istanbul University Clinical Research Ethics Committee (13 August 2018, 2018/1052). Written informed consent was obtained from the parents or legal guardians of all participants. All procedures were performed in accordance with the ethical standards of the Declaration of Helsinki.

## 3. Results

Demographic characteristics and antibody concentrations of the study cohort are presented in Table 1. A total of 16 patients were included, with a mean age of 11.8 ± 3.3 years (range: 5.5–16.5); 8 (50%) were female and 8 (50%) were male. There were no statistically significant differences between the patient and control groups in terms of age or sex (*p* = 0.76, 1.00, respectively). There were no statistically significant differences in the mean antibody titers of anti-HbsAg, anti-PCP, or anti-measles between the study and control groups (*p* = 0.74, 0.17, 0.93).

According to seroprotective antibody levels mentioned study design; The percentage of patients whose had protective antibody levels for anti-HbsAg and anti-PCP was 68.8% (n = 11), 100% (n = 16). For measles IgG, four (25%) patients were seronegative, three (18.8%) were equivocal, and nine (56.3%) were seropositive. According to the WHO-defined cutoff (<120 mIU/mL), two (12.5%) patients were considered susceptible to measles infection.

The baseline, 3rd and 6th month mean antibody titers for anti-HbsAg, anti-PCP, and anti-measles are shown in Table 2. At baseline, mean antibody titers for anti-HBsAg, anti-PCP, and anti-measles did not differ significantly between the study and control groups (*p* = 0.19, 0.61 and 0.11, respectively). Confidence intervals (95% CI) were calculated for each mean antibody titer (anti-HBsAg, anti-PCP, and anti-measles) to better illustrate the variability of the data. These intervals are presented alongside the mean ± SD values in Table 2.

**Figure 1:**
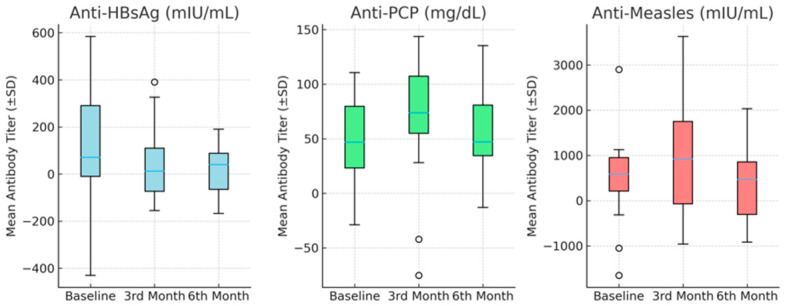
Box-and-Whisker Plots of Mean Antibody Titers in Children Receiving bDMARD Therapy (mean ± SD).

The distribution of antibody titers over time is illustrated in Figure 1, which presents box-and-whisker plots of mean antibody titers (mean ± SD) for anti-HBsAg, anti-PCP, and anti-measles in pediatric patients receiving bDMARD therapy.

The patients’ mean antibody titers and protective antibody levels at baseline at 3rd and 6th month are shown in Table 3. At the 3rd and 6th month follow-ups, none of the patients had anti-HbsAg protective titer levels below 10 mg/dl or anti-PCP level below 3.3 (Mg/L).

However, the anti-measles IgG titer decreased to below 200 mIU/mL in one patient (187 mIU/mL) in the 3rd month and in two patients (119 and 99 mIU/mL) in the 6th month. Three patients with a mean age of 14 ± 1.32 years, 2 (12.5%) male and 1 (6.25%) female, all of whom had juvenile idiopathic arthritis (JIA), received etanercept, and prior treatment was methorexate with prednisolone. No statistically significant differences were identified between patients whose titers decreased and those whose titers remained stable.

## 4. Discussion

In bDMARD therapy, although the degree of immunosuppression may vary among different drugs, patients who receive these drugs are at risk of serious bacterial and viral infections [8,11]. Infectious diseases remain a major concern in immunosuppressed children. The EULAR/PRES 2021 update emphasized the need for proactive vaccination strategies in pediatric rheumatic diseases [4]. Although routine childhood vaccination schedules and coverage vary among countries, in several studies, vaccination rates in children with pediatric rheumatological diseases have been found to be lower than those in healthy children [12]. In a study of children with JIA, every third patient was incompletely vaccinated, and another study reported that complete vaccination coverage in 52% of patients was lower than that in healthy controls [13,14]. In our study, all participants received hepatitis B, MMR, and PCV13 vaccines. Non-live vaccines, as noted in many studies, are safe and recommended for this patient group [4,12]. Our findings showed comparable mean antibody levels between patients and controls, but a subset of both groups lacked protective antibody titers for measles and hepatitis B at baseline. This observation highlights an important finding of the study—some fully vaccinated children exhibited low baseline antibody levels, suggesting waning immunity or an inadequate vaccine response despite full immunization.

The immunogenicity of the hepatitis B vaccine may be attenuated in patients receiving anti-TNF therapy, although relatively higher antibody responses have been observed with etanercept [15,16]. In our study, a mild decline in hepatitis B antibody titers was observed during follow-up. While not statistically significant, this trend has potential clinical relevance: booster vaccination may be warranted for children receiving long-term bDMARD therapy to maintain protective antibody levels.

PCV immunogenicity was measured using post-vaccination antibody titers against the serotypes of each vaccine. However, the protective titer varies according to age and pneumococcal disease [17]. Streptococcus pneumoniae is a leading human pathogen with high mortality, morbidity, and treatment costs. The EULAR/PRES recommends that those who are not vaccinated in this group, based on vaccine safety and immunogenicity studies, should be vaccinated with PCV10/13 [4]. In some studies, rituximab and MTX reduced the immunogenicity of pneumococcal vaccines, whereas other biologics (TNF- and IL-6 inhibitors) did not [16]. Children using anti-TNF had an equal seroprotective antibody compared with controls but had lower antibody concentrations in one study [18,19]. In our study, although the titers decreased in two patients after treatment, all titers were within the determined limits.

In a multicenter study, Uziel et al. demonstrated that live attenuated MMR booster vaccines were safe for children with rheumatic diseases who were receiving immunosuppressive therapies (including MTX and other biologics) [20]. In a study, it was shown that fully vaccinated children on anti-TNFα treatment maintained seroprotection rates and antibody titers against but detected accelerated antibody loss for measles compared with the control [21]. On the other hand, in another study, no difference was observed in anti-measles antibody levels compared to the control group [6]. In patients with pediatric rheumatological disease, there may be a decrease in disease-related or drug-related antibody titers [22], and all vaccine-specific memory B cells may be preserved in patients with a measles booster [23]. In our study, we detected decreases in antibodies with seroprotective levels in one patient at the 3rd month and in two patients at the 6th month. Notably, genetic factors may also contribute to reduced measles antibody persistence. HLA polymorphisms affecting measles vaccine-induced immunity have been reported which could partly explain interindividual variability in seroprotection [24].

This may help explain why a subset of fully vaccinated children in our study showed low baseline antibody titers. However, because of the short follow-up period, it was difficult to evaluate antibody decline significantly. Similarly, although the MMR vaccine maintains its immunogenicity for a long time in children with JIA who mostly receive anti-TNF, protective antibody titers have been shown to decrease over time in those receiving bDMARDs [25]. Surprisingly, there were cases of low baseline antibody levels in both the control and the patient groups that were fully vaccinated. In our study, there were cases of low hepatitis B and measles antibody titers before bDMARDs.

This study has several limitations, including its single-center design, small sample size, and short follow-up duration, which limit the generalizability of the results. The cohort included mainly JIA patients, and prior methotrexate or corticosteroid exposure may have influenced immune responses. Another limitation of this study was that follow-up sampling could not be performed in the control group. Although additional blood collection was initially planned, only a few participants returned for follow-up, and the limited number of samples was not sufficient for a meaningful statistical comparison.

## 5. Conclusions

Our findings highlight that antibody levels may be suboptimal even before the initiation of bDMARD therapy. Therefore, screening antibody status before and during treatment is essential to identify children at risk for inadequate vaccine-induced protection. Regular monitoring and timely booster vaccination should be considered to sustain adequate immunity and reduce the risk of vaccine-preventable infections in pediatric patients undergoing immunosuppressive therapy. Larger, multicenter studies with longer observation periods are needed to evaluate long-term vaccine-induced immunity in children receiving bDMARDs.

## Figures and Tables

**Table 1:** Characteristics of patient and control group demographics and a comparison of antibody titers.

Characteristics	Patients No. (%)	Control No. (%)	*p*
Age (y)			
5–11	8 (50)	11 (55)	
12–18	8 (50)	9 (45)	0.76
Sex			
Female	8 (50)	10 (50)	1.00
Male	8 (50)	10 (50)	
Diagnosis			
Jia	11 (68.8)		
Uveitis	5 (31.3)		
Pre-treatment			
Mix ^	8 (50)		
Mtx	4 (25)		
Other *	4(25)		
Treatment Adalimumab	7 (43.8)		
Etanercept	9 (56.3)		
Anti-HbsAg (mIU/mL)			
≥10	11 (68.8)	10 (50)	
<10	5 (31.3)	10 (50)	0.257
Anti-PCP(Mg/dL) 3.3–270	16 (100)	20 (100)	
Anti-Measles (mIU/mL)			
<150	4 (25)	6 (30)	
150–200	3 (18.8)	1 (5)	
>200	9 (56.3)	13 (65)	0.21
Susceptible (according to WHO)	2 (12.5)	5 (25)	
Duration of diseases (y, mean)	2.57 ± 2.26(0.25–7)		
Mean antibody titers (mean ± SD [95% CI])			
Anti-HbsAg	125.14 ± 290.56 [–29.66–279.94]	67.90 ± 124 [9.87–125.93]	0.74
Anti-PCP	59.10 ± 49.91 [32.51–85.69]	97.01 ± 93.01 [53.48–140.54]	0.178
Anti-Measles	641 ± 94 [4.77–1279.11]	389.63 ± 351.70 [225.03–554.23]	0.937

Jia: juvenile idiopathic arthritis, Mtx: methotrexate, * Nonsteroidal anti-inflammatory drug, colchicine or pd, ^ Mtx plus Prednisolone, y: year. 95% confidence intervals were calculated using the formula mean ± t (0.975, df) × (SD/√n). For patients, n = 16 (df = 15, t = 2.131); for controls, n = 20 (df = 19, t = 2.093). Because of the small sample size, the intervals are wide and should be interpreted with caution.

**Table 2:** Comparison of Mean Antibody Titers (mean ± SD, 95% CI).

	Baseline (Mean ± SD [95% CI])	3rd Month (Mean ± SD [95% CI])	6th Month (Mean ± SD [95% CI])	*p*
Anti-HBsAg (mIU/mL)	125.14 ± 290.56 [–29.66–279.94]	82.17 ± 166.44 [–6.50–170.84]	64.66 ± 118.66 [1.44–127.88]	0.19
Anti-PCP (mg/dL)	59.10 ± 49.91 [32.51–85.69]	61.80 ± 52.31 [33.93–89.67]	60.81 ± 50.43 [33.94–87.68]	0.61
Anti-Measles (mIU/mL)	641.94 ± 1196 [4.77–1279.11]	703.60 ± 1187 [71.23–1335.97]	585.22 ± 934 [87.63–1082.81]	0.11

95% confidence intervals were calculated using the formula mean ± t (0.975, df = 15) × (SD/√n). Because of the small sample size (n = 16), the intervals are wide and should be interpreted with caution.

**Table 3:** Mean antibody titers and protective antibody response of patients in baseline, 3rd and 6th months (mean ± SD, 95% Cl, n/%).

		Mean Antibody Titer	Protective Antibody Levels	Patients (No.%)
Anti-HbsAg (mIU/mL)	Baseline	125.14 ± 290.56 [−29.66–279.94]	≥10	11 (68.8)
			<10	5 (31.25)
	3rd *	82.17 ± 166.44 [−6.50–170.84]	≥10	11 (68.8)
			<10	5 (31.25)
	6th	64.66 ± 118.66 [1.44–127.88]	≥10	11 (68.8) *
			<10	5 (31.25)
Anti-PCP (Mg/dL)	Baseline	59.10 ± 49.91 [32.51–85.69]	3.3–270	16 (100)
	3rd	61.80 ± 52.31 [33.93–89.67]	3.3–270	16 (100)
	6th	71.44 ± 34.18 [33.94–87.68]	3.3–270	16 (100)
Anti-Measles (mIU/mL)	Baseline	641.94 ± 1196 [4.77–1279.11]	<150	4 (25)
			150–200	3 (18.75)
			>200	9 (56.25)
	3rd	703.60 ± 1187 [71.23–1335.97]	Decrease in titer (<200)	1 (6.25)
	6th	585.22 ± 934 [87.63–1082.81]	Decreases in titer (<200)	2 (12.5)

* Four patients had decreased titers compared to baseline but were still >10 mg/dL. 95% confidence intervals (CI) were calculated using the formula mean ± t (0.975, df = 15) × (SD/√n). Because of the small sample size (n = 16), the intervals are wide and should be interpreted with caution.

## Data Availability

The data presented in this study are available on request from the corresponding author. The data are not publicly available due to privacy or ethical restrictions.

## Supplementary Materials

The following supporting information can be downloaded at: https://www.mdpi.com/article/10.3390/children12111526/s1, Details of the commercial kits and analyzers used for antibody detection are provided in the Supplementary Material Table S1.

## Abbreviations

The following abbreviations are used in this manuscript:

bDMARD	biological disease-modifying antirheumatic drug
PCP	Pneumococcal capsular polysaccharide antigen
EULAR	The European League Against Rheumatism
MMR	Measles, Mumps, Rubella
WHO	World Health Organization
JIA	Juvenile idiopathic arthritis
PCV13	Pneumococcal 13-valent conjugate vaccine
MTX	Methotrexate
Anti-TNF	Anti-Tumor Necrosis Factor
PRES	Pediatric Rheumatology European Society
