# Supplementary material for: Preliminary Case–Control Study of Antibody Response to Vaccines in Children on bDMARDs
Source: Children (Basel). 2025 Nov 11;12(11):1526. doi: 10.3390/children12111526 (PMC12651895; doi:10.3390/children12111526)
Supplement: Supplementary file 1 [file children-12-01526-s001.zip › children-3900537-supplementary.pdf]

Supplementary Material

Table S1. Details of ELISA kits and analyzers used for antibody detection

Antibody	Assay Type / Kit	Manufacturer	Country	Assay Sensitivity / Specificity	Instrument Used
Anti-HBs (Hepatitis B)	Quantitative Chemiluminescent Microparticle Immunoassay (CMIA)	Abbott Diagnostics	USA	>99% / 98%	ARCHITECT i2000SR Immunoassay Analyzer
Anti-Measles IgG	Enzyme-Linked Immunosorbent Assay (ELISA)	Virion/Serion GmbH	Germany	Sensitivity >99%, Specificity 95%	TRITURUS® Automated ELISA System (Grifols)
Anti-PCP IgG (Pneumococcus)	VaccZyme™ Anti-PCP IgG Enzyme Immunoassay	Binding Site Group Ltd.	UK	Manufacturer-validated	TRITURUS® Automated ELISA System (Grifols)

Notes:

• Serum samples were stored at −20 °C until analysis.

• All assays were performed according to the manufacturers' instructions.

• The limit of quantification for the anti-PCP assay was 3.3–270 mg/dL.

• Protective antibody thresholds were defined as follows: ≥10 mIU/mL for anti-HBs, >120 mIU/mL for measles, per WHO 3rd International Standard (NIBSC 97/648).
